# Incubator-based active noise control device: comparison to ear covers and noise reduction zone quantification

**DOI:** 10.1038/s41390-023-02708-w

**Published:** 2023-07-06

**Authors:** George M. Hutchinson, Preston S. Wilson, Scott Sommerfeldt, Kaashif Ahmad

**Affiliations:** 1 Invictus Medical, Inc., San Antonio, TX USA; 2 Mechanical Engineering, University of Texas at Austin, Austin, TX USA; 3 Department of Physics and Astronomy, Brigham Young University, Provo, UT USA; 4 Pediatrix Neonatology of Houston, Houston, TX USA

## Abstract

**Background:**

Noise exposure in the neonatal intensive care unit (NICU) is consistently higher than current recommendations. This may adversely affect neonatal sleep, weight gain, and overall health. We sought to evaluate the effect of a novel active noise control (ANC) system.

**Methods:**

An ANC device’s noise reduction performance was compared to that of adhesively affixed foam ear covers in response to alarm and voice sounds in a simulated NICU environment. The zone of noise reduction of the ANC device was quantified with the same set of alarm and voice sounds.

**Results:**

The ANC device provided greater noise reduction than the ear covers in seven of the eight sound sequences tested in which a noise reduction greater than the just noticeable difference was achieved. For noise in the 500 Hz octave band, the ANC device exhibited consistent noise reduction throughout expected patient positions. It provided better performance for noise below 1000 Hz than above 1000 Hz.

**Conclusions:**

The ANC device provided generally superior noise reduction to the ear covers and provided a zone of noise reduction throughout the range where an infant would be placed within an incubator. Implications for patient sleep and weight gain are discussed.

**Impact:**

- Active noise control device can effectively reduce noise inside an infant incubator due to bedside device alarms.
- This is the first analysis of an incubator-based active noise control device and comparison to adhesively affixed silicone ear covers.
- A non-contact noise reduction device may be an appropriate means of reducing noise exposure of the hospitalized preterm infant.

## Introduction

Noise exposure in the neonatal intensive care unit (NICU) has been demonstrated to be consistently louder than the recommended guidelines of 45 dBA established by the American Academy of Pediatrics (AAP),^1–5^ which can interfere with patients’ sleep hygiene^6–9^ and affect their weight gain.^10,11^ Various approaches have been attempted to address this noise problem, including supplemental mechanical barriers to augment incubator walls, sound-triggered warning lights, and improved staff training; however, these attempts have not proven consistently effective.^12–14^ Single-family rooms are becoming more prevalent^15^ and have been shown to reduce noise levels, but still have average equivalent noise levels (Leq) ranging from 5 to 14 dB above AAP guidelines,^16^ and may be associated with adverse outcomes related to less infant interactions.^17^

NICU noise levels have been shown to impact the sleep hygiene of hospitalized infants.^6–9^ Preterm infants spend more time in quiet sleep compared to active sleep and are awake or fussy/crying less when wearing ear covers.^7,9^ Periods of sound changes of as little as 5–10 dB are associated with the transition from sleep to an awake state in very preterm infants, estimated to occur an average of 55 times per day.^8^ Sleep is one of the most important behavioral states for neonates, particularly preterm neonates,^18^ and sleep has been demonstrated to be critical for newborn development.^19^ Because the neonatal period coincides with a period of rapid brain development,^20^ the protection of infant sleep is critical to the care of NICU patients. Sleep and sleep cycles are essential for the development of the neurosensory and motor systems in the fetus and neonate, and the disruption of sleep and sleep cycles can significantly interfere with the early processes of sensory and brain development.^19–21^

NICU noise levels have also been linked to slower growth rates for preterm patients.^10,11^ Poor growth in preterm infants is associated with worsened long-term outcomes, including impaired neurodevelopment.^22–24^

Ear covers have been reported to provide improvements to both sleep hygiene and weight gain in incubated, preterm NICU patients.^6,7,9–11^ We sought to measure the level of noise reduction provided by commercially available neonatal ear covers and to compare this to that provided by a novel noise reduction device designed for use in neonatal incubators in a simulated NICU environment.^25^

## Materials and methods

### Study setting and measurement equipment

Experiments were conducted in the NICU simulation laboratory at The Children’s Hospital of San Antonio (San Antonio, Texas) using bedside critical care equipment consisting of the frequencies representative of a NICU, being more prominent in the 500 and 1000 Hz octave bands.^26^ The neonatal critical care equipment was arrayed around an infant incubator (Giraffe OmniBed, GE Healthcare, Waukesha, Wisconsin) in a typical manner. Alarm volumes were set to clinically appropriate levels. No patient consent was required in this study since no human subjects were used.

A microphone-equipped manikin weighted and sized to resemble a 1.3 kg 29-week gestational age preterm infant^27^ was used to collect sound data. The manikin was equipped with two general-purpose array microphones (Model 40PP, GRAS Sound and Vibration A/S, Holte, Denmark) embedded in the manikin head, each microphone having its sensing element positioned at an opening in the manikin’s molded ear. The microphones were interfaced to a computer equipped with the LabVIEW^TM^ Development System with the Sound and Vibration Toolkit (National Instruments, Austin, Texas) via a CompactDAQ Chassis containing a Sound and Vibration Input Module (National Instruments, Austin, Texas).

Using the critical care equipment and recorded voices, 11 sound sequences were used in testing (see Table 1) The critical care equipment included a patient monitor (IntelliVue® MX450, Philips Healthcare, Andover, Massachusetts), a syringe pump (Medfusion™ 2001, Smiths Medical, Minneapolis, Minnesota), a bubble continuous positive airway pressure ventilatory support device (Fisher Paykel, Auckland, New Zealand), and a ventilator (Maquet Servo-I, Getinge Group, Gothenburg, Sweden). These devices and the hospital air handling system were used to generate 11 clinically realistic sound sequences for testing that are primarily tonal. A male and a female voice were included in the sound sequences representing primarily non-tonal signals. These were recorded on a digital recorder (Zoom H4n, Zoom North America, Hauppauge, New York) via an externally connected microphone (Dayton Audio EMM-6, Dayton Audio, Springboro, Ohio). Recordings were 44.1 kHz 16-bit WAV files. The WAV files were replayed through a powered studio monitor (KRK Rokit 5, Gibson Pro Audio, Chatsworth, California). Recorded voices were used to ensure a consistent signal for all trials and conditions.

**Table 1:** Sound sequences used in performing testing.

No.	Device	Brand	Alarm priority	Primary tonal signal freq (Hz)	Secondary tonal signal freq (Hz)
Single device alarms					
1	Patient monitor	Philips	Medium	1440	480
2	Patient monitor	Philips	High	960	2880
3	Ventilator	Maquet	Medium	400	1173
4	Ventilator	Maquet	High	400	1173
5	Syringe pump	Medfusion	Low	2745	–
6	Syringe pump	Medfusion	High	2761	–
Multiple device simultaneous alarms					
7	(1) Syringe pump (2) Ventilator	Medfusion Maquet	High High	2761 400	– 1173
8	(1) Syringe pump (2) Ventilator	Medfusion Maquet	Low High	2745 400	– 1173
9	(1) Syringe pump (2) Patient monitor	Medfusion Philips	High Medium	2761 1440	– 480
Worst case scenario					
10	(1) Syringe pump (2) Ventilator (3) Patient monitor	Medfusion Maquet Philips	High High High	2761 400 960	– 1173 2880
Voice					
11	Male and female voice	N/A	N/A	No prominent tones	

All sound sequences are 60 s in duration.

**Fig. 1:**
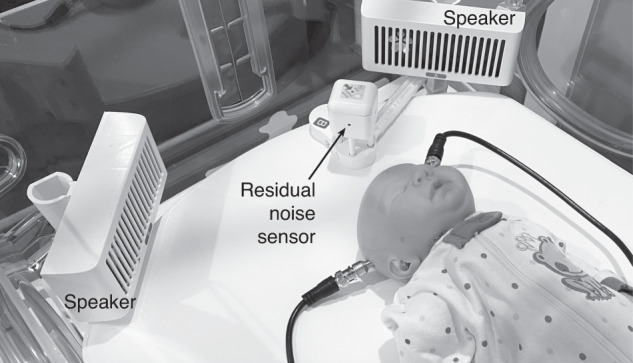
Elements of the ANC device placed inside the incubator. The ANC device includes two speakers and a residual noise sensor placed within the incubator.

Active noise control (ANC) was provided by a Neoasis® (Invictus Medical, San Antonio, Texas) ANC device, which was deployed on the infant incubator. The ANC device consists of a control unit and an outside noise sensor, both positioned outside the incubator, and two speakers and a residual noise sensor (RNS) positioned within the incubator and has been described previously^25^ (Fig. 1). Utilizing the phenomenon of incident wave summing, an ANC system generates a sound wave that is out of phase with an environment sound wave, resulting in the waves canceling each other, typically more efficiently at lower frequencies.^28^ The ANC device measures the sound waves outside the incubator, models what the sound waves will be after passing through the incubator wall, and generates a sound wave out of phase with the modeled wave. The RNS within the incubator provides data for the system to converge on an optimum solution.

The outside noise sensor was affixed to the incubator on the side closest to the primary noise source, which was the alarm or voice sound in each scenario. The speakers were mounted to small posts in the D-holes found in the corner of the incubator. The RNS, the two speakers, and the outside noise sensor were connected to the system’s control unit. No part of the ANC device contacted the manikin.

### ANC and passive ear cover comparison test method

**Fig. 2:**
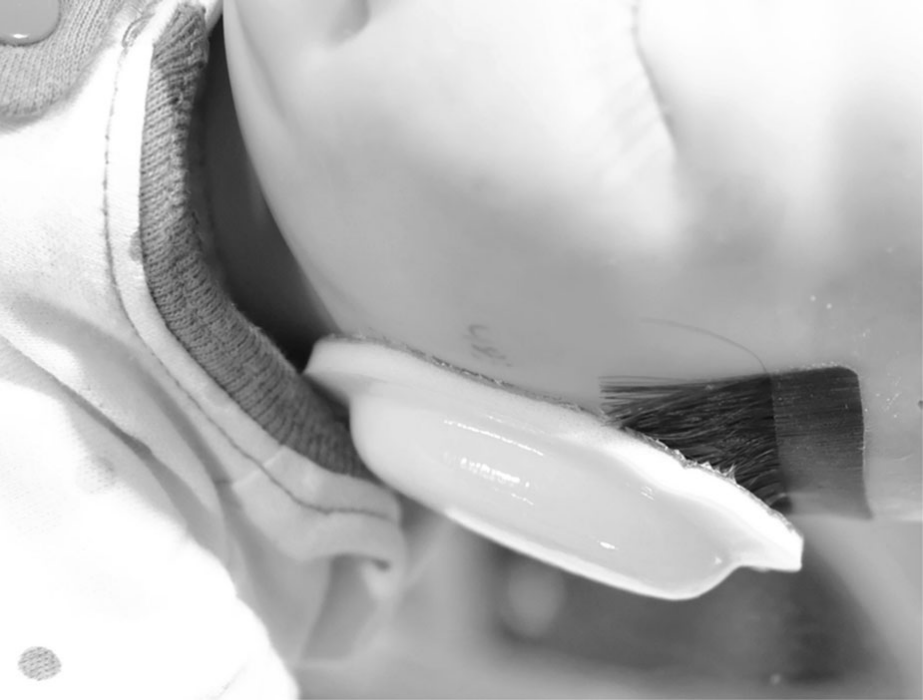
Ear covers adhered to manikin with hair extensions. Test conditions included ear covers adhered to the manikin over the auricles and adhered to the manikin over hair extensions that simulate the hair present in many infants.

In the first of the two protocols, the noise reduction efficiency of the ANC device was compared to that provided by foam ear covers adhered to the skin around each ear of the patient (MiniMuffs®, Natus Medical, Inc.). For each sound sequence in Table 1, sound pressure measurements were made at each manikin ear under four test conditions including (1) no noise reduction (control), (2) ANC device, (3) ear covers without hair, and (4) ear covers positioned on hair. Measurements under the four conditions were repeated five times in random order and the manikin was replaced in the incubator between each measurement. In normal use ear covers are applied to the head of an infant, covering the auricles, and therefore may be placed over the infant’s hair.^7^ Thus a test condition was included in which the ear cover is placed over hair. The hair used was a human hair extension cut to a length of 1 cm and adhered bilaterally above the manikin’s ears (see Fig. 2). The manikin was placed 5 cm from the RNS per instructions for use of the ANC device.

For each measurement, the standard octave bands from 250 to 8000 Hz for each test condition were adjusted with an A-weighting filter to account for the relative loudness perceived by the human ear. Octave bands were calculated with the “poctave” function of MATLAB (The MathWorks®, Natick, Massachusetts), which follows the ANSI S1.11 standard.^29^ The noise reduction provided by the ANC device and the ear covers with and without hair were calculated by subtracting those measured band levels from the control measurement band levels for each octave band. Between the right and left ears, results for the ear with the louder control band level were reported.

### Quantifying noise reduction zone

**Fig. 3:**
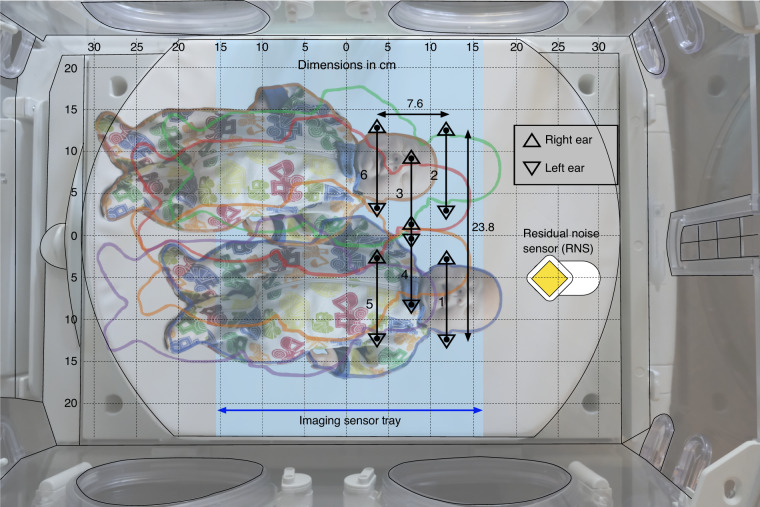
Position of the manikin in the incubator. An outline of the manikin is shown for positions 1 through 6 with a faint image of the manikin shown at positions 1 and 6. Right and left ear positions are indicated by triangles as indicated. The position of the incubator’s imaging sensor tray under the mattress frame is indicated by the light blue rectangle. All dimensions are in cm.

In the second of the two protocols, the effect of patient position on noise reduction efficiency was evaluated by measuring noise reduction at both ears of a manikin in six positions within an incubator with each sound sequence in Table 1. The positions of the manikin are shown (Fig. 3). Instances are noted when the resulting sound is greater than the recommended guidelines of 45 dBA^30^ and the ANC device has amplified the sound pressure level (SPL) by more than the just perceivable change (3 dB).^31^ The just noticeable difference is defined as the change in sound level that is just perceptible by normal hearing observers.^31^

Following the analysis of the data from the first protocol, which revealed the tonal nature of the test sequences, the analysis for this protocol was changed to focus on the primary tones of each signal. For each sound sequence and each position, A-weighted sound pressure measurements were made at each ear (as described in the previous section) with the ANC device off (control) and ANC device on. Spectra of each signal were calculated using the MATLAB function “pspectrum” with a resolution bandwidth of 6.25 Hz. Local maxima of at least 32 dBA, a level below which would be of no clinical concern, were identified with the SciPy package “find_peaks.” The noise reduction at each of these frequencies was then calculated as the dB difference between the ANC on and ANC off cases.

To quantify the relationship between frequency, position of the manikin, and noise reduction, the average noise reduction for all peaks in the 500, 1000, and 2000 Hz octave bands was calculated at each of the six physical measurement locations in the incubator.

## Results

### Comparison of noise reduction associated with ANC device and ear coverings

Noise reduction greater than 3 dB^31^ was provided by at least one of the noise reduction methods in eight of the 11 sound sequences (Table 2) ranging from 11.7 to 3.3 dB. For reference, a 20 dB reduction equals a 10-fold reduction in sound pressure and a 6 dB reduction equals a two-fold reduction in sound pressure. Of these eight, the ANC device provided the greatest noise reduction for seven sound sequences. The one sound sequence in which the ear cover provided the greatest noise reduction was for the recorded voice, at just a greater than noticeable difference. In this instance, the SPL was reduced from 36.7 to 32.0 dBA, a noise reduction of 4.7 dB (just less than a two-fold reduction). The ear covers positioned over hair did not offer the greatest noise reduction level for any sound sequence.

**Table 2:** Best attenuator among ANC device, ear cover without hair, ear cover over hair.

No.	Sound sequence	Brand	Alarm priority	Octave band (Hz)	Attenuation (dB, fold reduction)		
					Ear covers	Ear covers over hair	ANC device
2	Patient monitor	Philips	High	1000	3.4, 1.5	1.2, 1.1	11.7, 3.8^a^
3	Ventilator	Maquet	Medium	500	7.5, 2.4	2.5, 1.3	9.4, 3.0^a^
4	Ventilator	Maquet	High	500	2.6, 1.3	1.4, 1.2	8.6, 2.7^a^
7	(1) Syringe pump (2) Ventilator	Medfusion Maquet	High High	500	4.4, 1.7	0.6, 1.1	7.0, 2.2^a^
10	(1) Syringe pump (2) Ventilator (3) Patient monitor	Medfusion Maquet Philips	High High High	500	5.0, 1.8	5.4, 1.9	6.5 2.1a
8	(1) Syringe pump (2) Ventilator	Medfusion Maquet	Low High	500	2.4, 1.3	1.1, 1.1	5.1, 1.8^a^
11	Male and female voices	N/A	N/A	500	4.7, 1.7^a^	1.0, 1.1	0.1, 1.0
9	(1) Syringe pump (2) Patient monitor	Medfusion Philips	High Medium	500	2.7, 1.4	1.6, 1.2	3.3, 1.5^a^
1	Patient monitor	Philips	Medium	500	2.0, 1.3^a^	2.0, 1.3	0.1, 1.0
6	Syringe pump	Medfusion	Low	500	2.0, 1.3^a^	1.1, 1.1	0.1, 1.0
5	Syringe pump	Medfusion	High	500	1.5, 1.2^a^	1.1, 1.1	0.1, 1.0

The octave band column represents the octave band with the greatest attenuation value. The table is sorted from the most attenuation to the least.

^a^Best attenuation for each sound sequence.

**Fig. 4:**
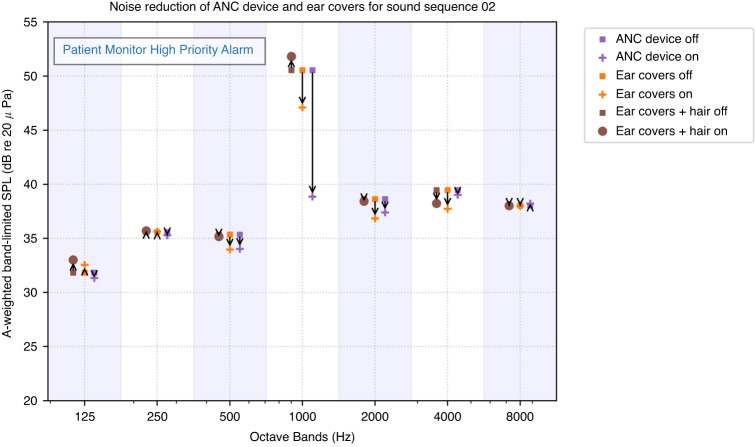
Effects of the three noise reduction methods on the octave band power spectrum for a high-priority patient monitor alarm. The octave bands for all octave bands from 125 to 8000 Hz are shown for the control condition (no noise reduction device) and each of the three noise reduction methods: ANC device, ear cover with no hair, and ear cover over hair.

An example of the noise reduction for a sound sequence is shown in Fig. 4. The alarm in this sound sequence is a patient monitor high-priority alarm with a tonal signal (consisting of a limited number of discrete tones as opposed to broadband noise) and has its primary frequency within the 1000 Hz octave band. The ANC device reduced the level in this band essentially down to the level of background noise in other octave bands, from 47.3 to 35.6 dBA, a noise reduction of 11.7 dB. The ear covers reduced the level in this band from 50.5 to 47.1 dBA, a noise reduction of 3.4 dBA. Ear covers over the hair increased the level in this band from 50.5 to 51.8 dBA, an amplification of 1.3 dB.

For syringe pump alarms having a primary frequency greater than 2000 Hz (sound sequences 5 and 6), none of these test conditions provided noise reduction greater than 3.0 dB but no octave band reached 39 dBA within the incubator. Among all test conditions, the only amplification greater than 3.0 was 4.5 dB, generated by the ear covers over the hair. The ear cover was noticed to have begun to peel off the manikin where it was placed over the hair.

In an unexpected finding, we discovered that when the ear cover’s adhesive begins to peel off the infant, the ear cover amplified the sound at the ear rather than attenuating it. Considering instances of amplification across all octave bands, all sound sequences, and all devices, the maximum amplification occurred with the ear covers over hair in seven of the 11 sound sequences and the only instances of amplification greater than 2 dB occurred with the ear covers on hair. This amplification may be due to a sound-concentrating phenomenon wherein the gap between the rim of the ear covers and the infant head causes the ear covers shell to behave similar to cupping a hand behind an ear. Alternately, the gap in between the ear cover’s shell and the infant head may approximate a Helmholtz resonator, akin to blowing air across the opening of an empty bottle. A gap across the top curve of the shell of one of the ear covers resulted in a gap approximately 50 mm long and 2 mm wide, providing a Helmholtz resonance frequency of 2267 Hz. Evaluating the response of the ear covers over hair with a device alarm having a primary frequency of 2200 Hz (BD Alaris™ Pump Module, Becton, Dickinson and Company, Franklin Lakes, New Jersey) resulted in a 5.4 dB amplification (46.0–51.4 dBA) in the 2000 Hz octave band, averaged over five trials thus supporting the hypothesis that when the ear covers release from the head of the infant, they may act like a Helmholtz resonator and thereby amplify sound rather than attenuate it. This implies that the ear covers may have a risk beyond simply coming loose and becoming ineffective sound blocks.

### Quantifying noise reduction zone of ANC device

**Fig. 5:**
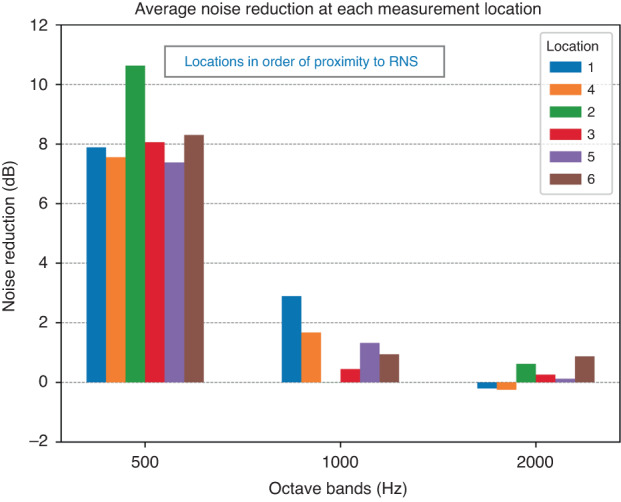
ANC device noise reduction at each location for each octave. The average noise reduction for the primary tonal signals of all sound sequences is shown for locations 1–6. For display in this figure, the tonal signals were grouped in octave band (500, 1000 or 2000 Hz) that includes the frequency of each tone. The order of locations 1–6 is arranged from closest to furthest from the residual noise sensor of the ANC device.

When using the ANC device, the average noise reduction across all sound sequences that possess primary frequencies in the 500 Hz octave band ranged from 7.4 to 10.7 dB at all positions tested within the incubator (Fig. 5). In the 1000 Hz octave band, the best average noise reduction was found in position 1, the position closest to the RNS (see Fig. 3) tailing off as the manikin was positioned further from the RNS. In the 2000 Hz octave band, little noise reduction was achieved.

**Fig. 6:**
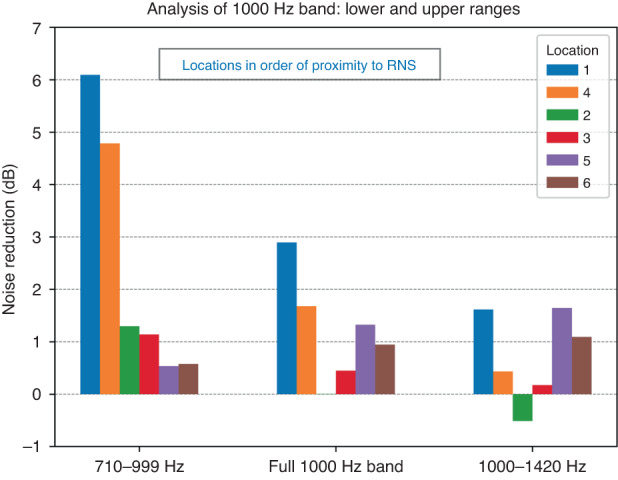
ANC device noise reduction at each location for 1000 Hz octave band. The bar graph shows the average noise reduction for all primary tonal frequencies falling within the 1000 Hz octave band, separating frequencies above and below 1000 Hz. The order of locations 1–6 is arranged from closest to furthest from the residual noise sensor of the ANC device. The combined responses in the entire 1000 Hz band are shown in the middle, the responses to the tonal signals below 1000 Hz are shown on the left, and the responses to the tonal signals above 1000 Hz are shown on the right.

Within the 1000 Hz band, noise reduction was better in the two positions closer to the RNS, as would be expected. A deeper look at this octave band shows that sounds with peaks in the spectrum below 1000 Hz were better attenuated than the sounds with spectral peaks above 1000 Hz (Fig. 6). In this instance, the two positions closest to the RNS show 6.1 and 4.8 dB noise reduction. With ANC devices, efficacy at lower frequencies is expected to be better than at higher frequencies.^28^

## Discussion

Exposure to excessive noise remains both problematic and persistent for infants requiring NICU hospitalization. Reductions in sound exposure using ear covers have been linked to improvements in sleep hygiene^7,9^ and weight gain, but reduced exposure to human voices and also are challenging to keep in place long term.^11^ In this work we demonstrate in a simulated setting, that a novel non-contact ANC device achieved better noise reduction for tonal signals such as those generated by electromechanical systems than the ear covers, without a need to apply the device directly to the infant and without concern for hair interfering with device application. This represents the first significant step toward introducing active noise cancellation for human use in the NICU setting.

Two prior studies investigated the effects of quieter environments for preterm NICU patients, comparing a control group exposed to standard-of-care conditions to a group of patients with ear covers adhered over their auricles.^7,9^ The ear cover-equipped group was associated with increased observations of quiet sleep compared with active sleep and fewer observations of awake periods using the Andersen Behavioral State Scoring system.

Demonstration of weight gain is an essential milestone for the successful discharge of NICU patients. Improved weight gain has been found in a group of patients fitted with ear covers compared to a control group of patients treated under standard care conditions.^11^ The better noise reduction found in this study with the ANC device when compared to the ear covers suggests improvements in weight gain may also be achievable with the ANC device. Earlier weight gain may be associated with earlier safe discharge, which has potential positive financial implications for managing healthcare costs.

Exposure to adult words and conversation turns are important for language developmental outcomes in NICU patients.^32,33^ The ANC device did not reduce the voice signal, which may be advantageous given the importance of adult voice exposure for the developing infant.^32,33^ Since ANC devices are more effective with sounds that are essentially periodic as is the case with machine-generated alarms, this technology is not generally effective on random sequence, non-tonal signals such as voice.^28^ While this reduction may be advantageous from the point of view of noise exposure, it may be disadvantageous as it relates to exposure to human voices for language development. To permit the full transmission of desired human voices, the ANC device tested has a mechanism to selectively transmit the parent’s or caregiver’s voice into the incubator and present the infant’s sounds in response. A microphone positioned within a control unit outside the incubator can be selectively activated with a control unit switch so that voice signals can be transmitted to the ANC device’s speakers inside the incubator, providing to the infant a voice signal unmuffled by the incubator walls. At the same time, the ANC device’s RNS provides an audible signal from the infant to a speaker positioned in the same control unit for the parent or caregiver to hear.

Neither the ear covers nor the ANC device provided significant noise reduction in the 2000 Hz octave band and above. However, the energy content that NICU alarms present within the infant incubator is typically lower at higher frequencies.^26^ Measurements of the unweighted octave band levels within an infant incubator in response to 11 bedside devices’ alarms found that compared to the level of the 500 and 1000 Hz octave bands, the level in the 2000 Hz band was 8 dB lower and the 4000 Hz band was 14 dB lower. This would imply that performance in the higher frequencies is less important for a noise control device.

This study has several potential limitations. Data were collected in a simulated NICU room located within a hospital simulation center. The bedside devices used were selected to cover the range of frequencies found in NICU devices.^26^ However, other devices having alarms with different temporal characteristics may elicit different noise reduction profiles. While the ANC device tested uses an adaptive algorithm that accommodates different acoustic environments (as would be found in differently shaped incubators) our testing was conducted only in a GE Healthcare Giraffe OmniBed incubator. Different incubators may result in different performances with the noise reduction methods evaluated. Although other research has found clinical benefits associated with a reduction in sound levels in the NICU similar to that provided by this ANC device, clinical benefits cannot be attributed to the use of this ANC device without a clinical trial. The manikin used in testing is generally soft but is not a human infant. Lastly, the ear covers tested may adhere differently to an infant’s skin compared to the surface of the manikin head.

In subsequent work, the ANC device should be evaluated in a clinical environment to validate whether the lowering of noise levels inside the incubator results in clinical benefits. Studies showing improvements in sleep hygiene^7,9^ and weight gain changes^10,11^ in a quieter NICU environment may provide templates for protocols and clinical endpoints. An assessment of the length of stay changes for matched cohorts of patients would also be a relevant study.

In conclusion, the ANC device provided superior noise reduction to the ear covers in this simulated environment test for all alarm sounds where the noise reduction difference was greater than the just noticeable difference. Adhesively affixed ear covers, when peeling off, can amplify sounds rather than attenuate them. These simulation results demonstrate for the first time, in principle, the ability to successfully utilize active noise cancellation in the NICU setting to reduce environmental noise exposure.

## Data Availability

The datasets generated during and/or analyzed during the current study are available from the corresponding author on reasonable request.
